# Impaired Autophagic Clearance with a Gain-of-Function Variant of the Lysosomal Cl^−^/H^+^ Exchanger ClC-7

**DOI:** 10.3390/biom13121799

**Published:** 2023-12-15

**Authors:** Shroddha Bose, Cecilia de Heus, Mary E. Kennedy, Fan Wang, Thomas J. Jentsch, Judith Klumperman, Tobias Stauber

**Affiliations:** 1 Institute for Molecular Medicine, MSH Medical School Hamburg, 20457 Hamburg, Germany; 2 Institute of Chemistry and Biochemistry, Freie Universität Berlin, 14195 Berlin, Germany; 3 Center for Molecular Medicine/Cell Biology, University Medical Center (UMC), 3584 CX Utrecht, The Netherlands; 4 Leibniz-Forschungsinstitut für Molekulare Pharmakologie (FMP), 13125 Berlin, Germany; 5 NeuroCure Cluster of Excellence, Charité Universitätsmedizin Berlin, 10117 Berlin, Germany

**Keywords:** autophagy, chloride/proton exchange, ClC-7, endo-lysosomes, lysosomal storage disorder, osmotic pressure, Ostm1

## Abstract

ClC-7 is a ubiquitously expressed voltage-gated Cl^−^/H^+^ exchanger that critically contributes to lysosomal ion homeostasis. Together with its β-subunit Ostm1, ClC-7 localizes to lysosomes and to the ruffled border of osteoclasts, where it supports the acidification of the resorption lacuna. Loss of ClC-7 or Ostm1 leads to osteopetrosis accompanied by accumulation of storage material in lysosomes and neurodegeneration. Interestingly, not all osteopetrosis-causing *CLCN7* mutations from patients are associated with a loss of ion transport. Some rather result in an acceleration of voltage-dependent ClC-7 activation. Recently, a gain-of-function variant, ClC-7^Y715C^, that yields larger ion currents upon heterologous expression, was identified in two patients with neurodegeneration, organomegaly and albinism. However, neither the patients nor a mouse model that carried the equivalent mutation developed osteopetrosis, although expression of ClC-7^Y715C^ induced the formation of enlarged intracellular vacuoles. Here, we investigated how, in transfected cells with mutant ClC-7, the substitution of this tyrosine impinged on the morphology and function of lysosomes. Combinations of the tyrosine mutation with mutations that either uncouple Cl^−^ from H^+^ counter-transport or strongly diminish overall ion currents were used to show that increased ClC-7 Cl^−^/H^+^ exchange activity is required for the formation of enlarged vacuoles by membrane fusion. Degradation of endocytosed material was reduced in these compartments and resulted in an accumulation of lysosomal storage material. In cells expressing the ClC-7 gain-of-function mutant, autophagic clearance was largely impaired, resulting in a build-up of autophagic material.

## 1. Introduction

ClC-7 is a ubiquitously expressed electrogenic 2Cl^−^/H^+^ exchanger on lysosomes [1,2,3]. In addition to its lysosomal localization, ClC-7 is also found on the ruffled border of osteoclasts that is formed by lysosomal exocytosis [1,4]. ClC-7 requires a small type-I transmembrane protein, Ostm1, as a β-subunit for stability and ion transport [3,5]. The heavily glycosylated luminal N-terminus of Ostm1 is thought to protect ClC-7 from proteolytic degradation in the lysosomal lumen [5,6,7]. Mutations in either ClC-7 or Ostm1 result in osteopetrosis in human patients and animal models [1,8]. This is additionally accompanied by an accumulation of lysosomal storage material and neurodegeneration with features of ceroid lipofuscinosis in animal models [5,9,10] and in a subset of patients [11,12]. Accordingly, loss of ClC-7 is shown to impair lysosomal protein degradation [13]. This appears not to be due to a change in the crucial acidic luminal pH of lysosomes but rather to reduced pH gradient-driven Cl^−^ accumulation by ClC-7/Ostm1 [14,15]. Recently, it was shown that reduced lysosomal Cl^−^ indeed diminishes the activity of some lysosomal hydrolases [16,17].

Surprisingly, not all disease-causing *CLCN7* mutations lead to a loss of function. Rather, several mutations accelerate the voltage-dependent gating of ClC-7 [3,11,18,19]. Intriguingly, yet another type of gain-of-function mutation, ClC-7^Y715C^, was recently identified in two unrelated heterozygous patients with albinism, myelination defects and lysosomal storage but without apparent bone phenotype [20]. ClC-7^Y715C^ mediated ion currents with larger amplitudes, reportedly due to a lack of inhibition by phosphoinositide lipids [20,21]. Cells from the patients and from knock-in mice with the mutation in Tyr^715^ or the equivalent Tyr^713^ in murine ClC-7, respectively, displayed strikingly large non-acidic vacuoles, while smaller lysosomes in the same cells were reported to be hyperacidified [20].

In this study, we investigate how the gain-of-function mutation in this critical tyrosine of ClC-7 affects lysosomal morphology and function. We find that its effect on endo-lysosomal morphology requires the Cl^−^/H^+^ exchange activity of ClC-7. The enlargement of endo-lysosomal compartments is accompanied by altered lysosomal pH, reduced degradative capacity and defective autophagic clearance.

## 2. Materials and Methods

*Cell culture.* HeLa cells obtained from Leibniz Forschungsinstitut DSMZ (Deutsche Sammlung von Mikroorganismen und Zellkulturen) were maintained in DMEM growth medium supplemented with 10% FBS, 100 units/mL penicillin and 100 µg/mL streptomycin. U2OS cells stably expressing LAMP1-GFP, kindly provided by Ian Ganley, University of Dundee [22], were maintained with McCoy’s 5A medium in presence of 10% FBS and 1% penicillin–streptomycin. Both cell lines were kept at 37 °C in a humidified atmosphere with 5% CO_2_.

*Transient transfection.* Expression constructs of rClC-7 in vector pcDNA3 for expression in mammalian cells or vector pTLN for expression in *Xenopus* oocytes, as well as Ostm1 constructs, have been previously described [3,23]. New mutations were introduced by PCR and combined by restriction digest and ligation. All constructs were confirmed by sequencing the complete open reading frame. HeLa cells and U2OS LAMP1-GFP cells were co-transfected with ClC-7 and Ostm1 constructs at a 20:1 mass ratio using FuGENE 6 (Promega, Walldorf, Germany) according to the manufacturer’s instructions. Transfection medium was replaced with fresh medium after 7–8 h.

*Live cell imaging of vesicle formation and resolution.* The 9 × 10^4^–2 × 10^5^ U2OS LAMP1-GFP cells were plated on glass-bottom live-cell dishes (MatTek, Bratislava, Slovak Republic) and transfected with ClC-7^Y713F^/Ostm1-RFP using FuGENE 6. Thirty-six hours after transfection, cells were washed once with PBS, covered with live imaging buffer supplemented with 10% FCS and imaged with a Nikon-CSU spinning disk confocal microscope on a heating stage at 37 °C. LAMP1-GFP fluorescence was excited at 488 nm and detected at 535 nm. Images of live cells were taken at one frame per second.

*Immunostaining.* Transfected HeLa cells were fixed with 4% PFA in PBS for 15 min, permeabilized with antibody buffer (0.05% saponin and 3% BSA in PBS) for 15 min and subsequently incubated with primary and AlexaFluor-coupled secondary antibodies diluted in antibody buffer for 90 min and 60 min, respectively. Primary antibodies were rabbit anti-ClC-7 (7N4B [1], 1:200), mouse anti-LAMP1 (H4A3, Developmental Studies Hybridoma Bank (DSHB), The University of Iowa, Iowa City, IA, USA, deposited to the DSHB by August, J.T. / Hildreth, J.E.K., 1:250), mouse anti-Rab5 (Abcam, Cambridge, UK, ab18211, 1:250), rabbit anti-EEA1 (Abcam, ab2900, 1:500), Rab7a (Cell Signaling Technology, Leiden, The Netherlands, 1:500), mouse anti-LBPA (BD Pharmingen, Heidelberg, Germany, 1:250), mouse anti-CD63 (BD Pharmingen, 1:500), rabbit anti-LC3 (mbl, Minneapolis, MN, USA, 1:500) and mouse anti-p62 (Cell Signaling Technology, 1:500). Images were acquired with a Leica DMi8 light microscope (Leica Microsystems, Wetzlar, Germany) equipped with a 63x/1.40 NA oil-immersion lens and respective filter cubes (FITC for Alexa 488; TRITC for mRFP and Y5.5 for Alexa633).

*Immunoblotting.* Western blot of HeLa cell lysates (20 μg per lane) were probed with the following primary antibodies: guinea pig or rabbit anti-ClC-7 (7N4B [1], 1:500), rabbit anti-LC3 (mbl, 1:500), mouse anti-LAMP1 (H4A3, Developmental Studies Hybridoma Bank, deposited by August, J.T. / Hildreth, J.E.K., 1:200), mouse anti-p62 (Cell Signaling Technology, 1:500), goat anti-cathepsin D (C-20, Santa Cruz, Heidelberg, Germany, 1:500) and rabbit anti-GAPDH (14C10, Cell Signaling Technology, 1:2500). Horseradish peroxidase (HRP)-conjugated species-specific secondary antibodies (Jackson ImmunoResearch, Ely, UK) were used and immunoreactive signals were detected using an enhanced chemiluminescence reagent (HRP juice; PJK, Kleinblittersdorf, Germany) and a ChemiSmart5000 digital imaging system (Vilber-Lourmat, Eberhardzell, Germany). Densitometrical quantification was performed with the Fiji (2.0.1) software [24].

*Two-electrode voltage clamp.* Defolliculated oocytes from *Xenopus laevis* were injected with cRNA transcribed from pTLN vectors using the mMESSAGE mMACHINE kit (Invitrogen, Darmstadt, Germany). An amount of 23 ng of ClC-7^WT^ and ClC-7 mutant constructs along with 23 ng of Ostm1 cRNA were injected as previously described [3]. Following injection, the oocytes were kept for 2 days at 17 °C before currents were measured by two-electrode voltage clamp using TurboTEC amplifiers (npi electronic GmbH, Tamm, Germany) and pClamp 10 software (Molecular Devices, San Jose, CA, USA). ND96 saline (96 mM NaCl, 2 mM potassium gluconate, 1.8 mM calcium gluconate, 1 mM magnesium gluconate, 5 mM HEPES, pH 7.5) was used to superfuse the oocytes. Currents were generated by clamping the cells for 2 s to voltages between −100 mV and +80 mV in 20-mV steps, followed by a repolarizing step to −100 mV for 1 s. Leak and capacitive currents were not compensated for. Capacitive transients were removed from the figures for clarity. Time constants of activation and deactivation were obtained from single exponential fits to the current trace from 25 to 325 ms after the voltage step to 80 mV or to −100 mV following a 2 s pulse to 80 mV, respectively. Current amplitudes at 80 mV were normalized to the ones in standard ND96.

*Measurement of lysosomal pH.* Lysosomal pH was measured essentially as previously described [14]. Twenty-four hours post-transfection, HeLa cells were loaded with 0.5 mg/mL Oregon Green 488-dextran (Life Technologies, Darmstadt, Germany) in growth medium overnight, followed by a two hour chase. Cells were imaged in imaging buffer using a Leica Dmi8 microscope (Leica Microsystems) equipped with a 63x/1.40 NA oil-immersion lens and an Oregon Green filter cube (AHF, Tübingen, Germany) with excitation at 440 nm or 480 nm, respectively, delivered by an OptoScan monochromator (Cairn Research, Kent, UK) controlled by the microscopy software WinFluor (V3.8.7, University of Strathclyde, Glasgow, UK), and emission at 516–556 nm. In situ pH calibration curves were obtained for each dish in isotonic solutions supplemented with 10 μM nigericin and 10 μM monensin after equilibration for at least 2 min for each pH (ranging from pH 4 to 6.5) starting with pH 6.5. Images were analyzed using ImageJ with regions of interest (ROIs) around cells or individual enlarged vacuoles. Fluorescence intensity ratios (488/440) as a function of pH were fit to a sigmoid and used to interpolate the pH values from the cells prior to the calibration.

*LysoTracker assay.* U2OS LAMP1-GFP cells were co-transfected with ClC-7^Y713F^/Ostm1-RFP 14 h after plating on glass-bottom live-cell dishes (MatTek). Twenty-four hours after transfection, 50 nM LysoTracker Deep Red (Invitrogen) was applied to the cells for 30 min. Cells were subsequently washed and imaged immediately with live-cell imaging buffer, using a DMi8 microscope (Leica Microsystems), 63x/1.40 NA oil-immersion objective, OcraFlash 4.0 camera (Hamamatsu Photonics, Herrsching am Ammersee, Germany) at 16-bit, 2x2 binning using FITC, FI/TRITC, Y5.5 and DAPI filter set.

*Lysosomal cargo uptake assay.* HeLa cells were co-transfected with ClC-7^Y713F^/Ostm1-RFP 14 h after plating on glass-bottom live-cell dishes (MatTek). Lysosomes were loaded with 0.5 mg/mL of Alexa 488-dextran immediately after transfection. Six hours later, the medium was changed with fresh medium containing 0.5 mg/mL of Alexa 647-dextran. After a 16 h pulse, the cells were chased for 2 h with complete media, followed by washing and imaging of the cells in live imaging buffer.

*Lysosomal degradation assay.* Sixteen hours after plating, HeLa cells were co-transfected ClC-7^Y713F^/Ostm1-RFP on glass-bottom live-cell dishes (MatTek). Sixteen to eighteen hours after transfection, 10 µg/mL DQ Green BSA dye (DQ-BSA, Life Technologies) was applied to the cells for one hour at 37 °C. After rinsing with PBS, cells were imaged the following day. Similarly, 1 µM, BODIPY FL-pepstatin A conjugate (Life Technologies) was loaded on ClC-7^Y713F^/Ostm1-RFP co-transfected HeLa cells for 1 h at 37 °C, washed with PBS, incubated overnight and imaged the following day with a DMi8 microscope (Leica Microsystems), 63x/1.40 NA oil-immersion objective, OcraFlash 4.0 camera (Hamamatsu Photonics) at 16-bit and 2x2 binning using FITC and FI/TRITC filter sets.

*Electron microscopy.* For preparing samples for Epon transmission electron microscopy (TEM) [25], HeLa cells were co-transfected with ClC-7^Y713F^/Ostm1-GFP and ClC-7^WT^/Ostm1-GFP 12 h after plating on 35 mm dishes along with untransfected HeLa cells. Twenty-four hours after transfection, the cells were fixed with half-strength Karnovsky fixative (2% para-formaldehyde (PFA), 2.5% glutaraldehyde (GA) in 0.1M phosphate buffer, pH7.4) for two hours at room temperature. After scraping, cells were pelleted and embedded in 2% low melting point agarose. Cell pellets were post-fixed with 1% OsO_4_, 1.5% K_3_[Fe(CN)_6_] in 0.065 M Na-cacodylate, stained en bloc with 0.5% uranyl acetate (UA), dehydrated in acetone and embedded in Epon. Ultrathin sections were stained with uranyl acetate and lead citrate for 2 h at room temperature.

For immunoelectron microscopy (immuno-EM), HeLa cells were co-transfected with ClC-7^Y713F^/Ostm1-GFP and ClC-7^WFT^/Ostm1-GFP 12 h after plating on 35 mm dishes along with untransfected HeLa cells. Twenty-four hours after transfection, the cells were fixed by adding 4% PFA in 0.1 M phosphate buffer to an equal volume of medium. Alternatively, cells were fixed in 2% PFA and 0.2% GA. Post-fixation was carried out overnight at 4 °C using 4% PFA in 0.1 M phosphate buffer, after which cells were stored in 1% PFA at 4 °C. Cells were prepared for cryosectioning and immunogold labeling as previously described [26]. In short, fixed cells were scraped from the culture dishes and collected in PBS with 1% gelatin. After cells were pelleted, 1% gelatin was replaced by 12% gelatin at 37 °C and cells were pelleted again. The pellets were solidified on ice, cut into smaller blocks and infused with 2.3 M sucrose overnight (ON) at 4 °C. The smaller blocks were mounted on pins and stored in liquid nitrogen until cryosectioning into 90 nm thick sections at −100 °C on a DiATOME diamond knife in a Leica ultracut cryomicrotome. Sections were picked up and deposited on formvar- and carbon-coated grids using 2.3 M sucrose and 1.8% methylcellulose (MC) mixed 1:1. Sections were incubated in PBS at 37 °C for ~30 min to remove the gelatin mixture. After washing and blocking, sections were labeled with the following primary antibodies: goat anti-GFP (Rockland, Heerhugowaard, The Netherlands), mouse anti-LAMP1 (H4A3, BD Pharmingen) or mouse anti-p62 (3/p62 lck ligand, BD Transduction Laboratories), followed by bridging antibodies rabbit anti-mouse IgG (Rockland) or rabbit anti-goat IgG (Nordic-MUbio, Susteren, The Netherlands). Grids were then incubated with Protein A conjugated to 10–15 nm gold particles (Cell Microscopy Core, UMC Utrecht, The Netherlands), postfixed for 5 min using 2% UA, pH 7.0, followed by UA/MC mixture, pH 4.0, for 10 min at 4° C. Imaging was performed on a Tecnai T12 TEM using serialEM software [27] (University of Colorado, Boulder, CO, USA; generated by Nexperion, Vienna, Austria, based on version 1.2.16).

## 3. Results

### 3.1. Enlargement of Lysosomes by Tyr713 rClC-7 Mutant Depends on Presence of Ostm1

**Figure 1:**
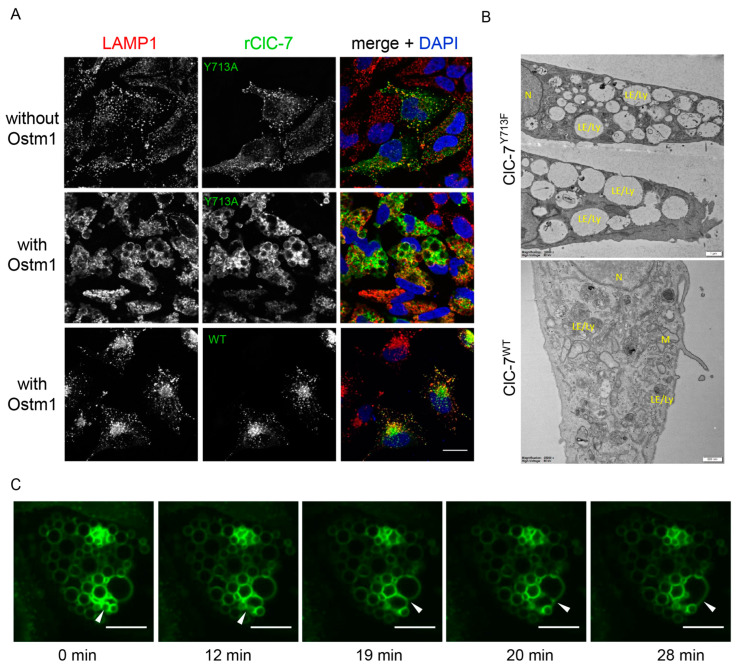
Alteration of lysosomal morphology upon mutation of a tyrosine residue of ClC-7. (**A**) Representative images showing the effect of overexpression of ClC-7^Y713A^ in HeLa cells without and with co-expression of its β-subunit Ostm1. Immunostaining with LAMP1 and ClC-7 antibodies shows lysosomal localization of ClC-7 both in absence and in absence of Ostm1, but alteration of lysosomal morphology only in presence of Ostm1. Scale bar, 20 µm. (**B**) Representative electron microscopy images of Epon sections showing the morphology of endo-lysosomes in ClC-7^WT^ cells as variably shaped organelles with dense irregular content. By contrast, ClC-7^Y713F^/Ostm1-expressing cells show an accumulation of electron-lucent vacuoles with variable content. LE/Ly—late endosome/lysosome; M—mitochondria; N—nucleus. Scale bars, 0.5 µm for ClC7^WT^ and 1.0 µm for ClC-7^Y713F^. (**C**) Representative images from a time-lapse video of a LAMP1-GFP-expressing U2OS cell co-transfected with ClC-7^Y713F^/Ostm1 tracked over 30 min. White arrowheads indicate sites of vesicle fusion. Scale bar, 10 µm.

The ClC-7^Y715C^ mutation causes a vacuolization in cells from patients and mice [20]. In a previous study focused on the identification of endo-lysosomal sorting motifs of intracellular CLCs, we mutated this tyrosine (Tyr^715^ in human, Tyr^713^ in rat ClC-7), which was located within a presumed canonical YXXΦ sorting motif in the cytoplasmic C-terminal region of ClC-7. Substituting this tyrosine had no impact on the binding of sorting adaptor protein complexes to ClC-7, and ClC-7^Y713A^ localized normally to lysosomes upon heterologous expression, with no apparent morphological alterations of these organelles [23]. However, when ClC-7^Y713A^ (Figure 1A) or ClC-7^Y713F^ (not shown) were co-transfected with Ostm1, which is essential for ion transport by ClC-7 [3] without affecting its subcellular trafficking [5,23], this resulted in a dramatic enlargement of lysosomal compartments (Figure 1A). Since mutation of the tyrosine to alanine, cysteine or phenylalanine similarly affected lysosomal morphology, we used the Y713F mutation for further analysis because of the structural similarity between tyrosine and phenylalanine. Electron micrographs confirmed the presence of enlarged compartments in HeLa cells transfected with ClC-7^Y713F^/Ostm1 (Figure 1B). Notably, the vacuoles were not surrounded by smaller vesicles. To gain insight into the process driving the enlargement, we tested if the structures grew larger over time and if we could observe the expected vesicle fusion that must occur to provide membrane for the large vacuoles. To this end, we ectopically expressed ClC-7^Y713F^/Ostm1-RFP in U2OS cells stably expressing LAMP1-GFP and monitored the lysosomal marker LAMP1-GFP by live-cell confocal microscopy, revealing multiple fusion steps of LAMP1-positive vesicles to form enlarged vacuoles (Video S1 and Figure 1C).

### 3.2. Cl^−^/H^+^ Exchange Activity Is Required for the Enlargement of Lysosomes

**Figure 2:**
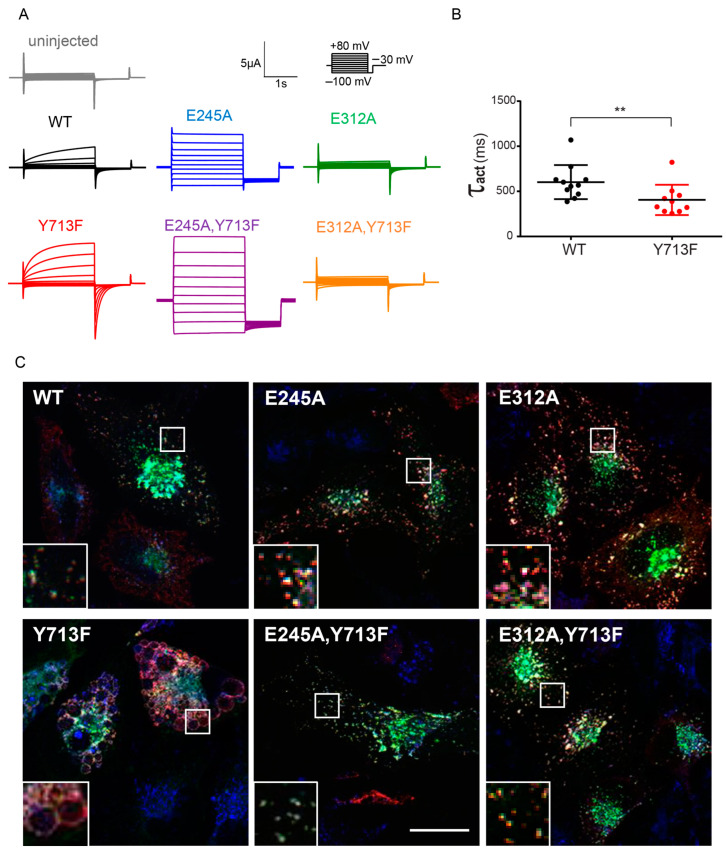
ClC-7 gain-of-function leads to enlargement of lysosomes. (**A**) Representative current traces from two-electrode voltage-clamp measurements of ClC-7/Ostm1-mediated currents in *Xenopus* oocytes (from at least 5 batches of oocytes) co-expressing Ostm1 and plasma membrane-localized ClC-7^PM^, carrying no further mutation (WT) or combinations of mutations E245A, E312A or Y713F, as indicated. Currents of uninjected oocytes serve as control. Protocol as shown in inset: holding potential −30 mV, followed by 2 s test pulses between −100 mV and +80 mV at 20 mV steps, each followed by a 1 s deactivation pulse at −100 mV. (**B**) Rate constants of current activation were determined by a single exponential fit of the current trace during the first 250 ms of depolarization to 80 mV for each measured oocyte. Thick lines in data point clouds indicate the arithmetic mean, thin lines the s.e.m. Values are mean of 11 (WT) and 10 (Y713F) oocytes from at least 3 batches of oocytes (Mann-Whitney test; significance shown as ** *p* < 0.005). (**C**) Representative images of immunostaining of HeLa cells 36 h after co-transfection with rClC-7 mutants and Ostm1-GFP (green), fixed and stained for ClC-7 (red) and LAMP2 (blue). Only ClC-7^Y713F^ shows a massive enlargement of lysosomal compartments. Scale bar, 20 µm.

To examine the effect of the tyrosine mutation on ion transport by ClC-7, we used a partially plasma membrane-localized ClC-7 mutant with disrupted N-terminal sorting motifs [23] expressed together with Ostm1 in *Xenopus* oocytes for two-electrode voltage-clamp measurements. As previously reported for the patient mutation Y715C [20], mutation of Tyr713 in rClC-7^Y713F^ increased outward currents by ClC-7 (Figure 2A), with a current amplitude of 0.50 ± 0.08 µA for ClC-7 and 1.09 ± 0.19 µA for ClC-7^Y713F^ at 60 mV. The mutation also moderately accelerated the voltage-dependent activation of ClC-7, with a time constant τ_act_ of 602 ± 57 ms for rClC-7^WT^ and 405 ± 53 ms for rClC-7^Y713F^ (Figure 2B). As in other CLC exchangers, mutation of a critical so-called ‘gating’ glutamate to alanine (E245A in rClC-7) uncouples chloride transport from proton countertransport by ClC-7. It also abrogates the slow voltage activation and the outward rectification, converting ClC-7 into a pure chloride conductance with near-ohmic currents [3,14]. When combined with the Y713F mutation, the ClC-7^E245A,Y713F^ double mutant displayed the same current properties, except for a larger amplitude (Figure 2A). Mutation of another critical glutamate, the ‘proton’ glutamate (E312 in rClC-7), strongly diminished ClC-7 currents [3,28], both in the plasma membrane-localized “WT” and in combination with the Y713F mutation in ClC-7^E312A,Y713F^ (Figure 2A).

Corroborating the notion that ion transport by ClC-7^Y713F^ is required for the enlargement of lysosomal compartments, co-expression of ClC-7^E312A,Y713F^ with Ostm1 did not lead to the enlargement of LAMP1-positive compartments, to which ClC-7^E312,Y713F^/Ostm1 and ClC-7^E312A^/Ostm1 [29] correctly localized (Figure 2C). Combination of the Y713F mutant with the E245A uncoupling mutation in the ClC-7^E245A,Y713F^ mutant also abrogated the generation of large LAMP1-positive structures despite the increased current amplitude by this mutant (Figure 2B) and its lysosomal localization (Figure 2C), which was expectedly not affected by the E245A mutation [14]. This suggests that the Cl^−^/H^+^ exchange activity of ClC-7/Ostm1 is required for the enlargement. It cannot be replaced by a pure Cl^−^ conductance.

### 3.3. Late Endosomal/Lysosomal Nature of the Enlarged Vacuoles

**Figure 3:**
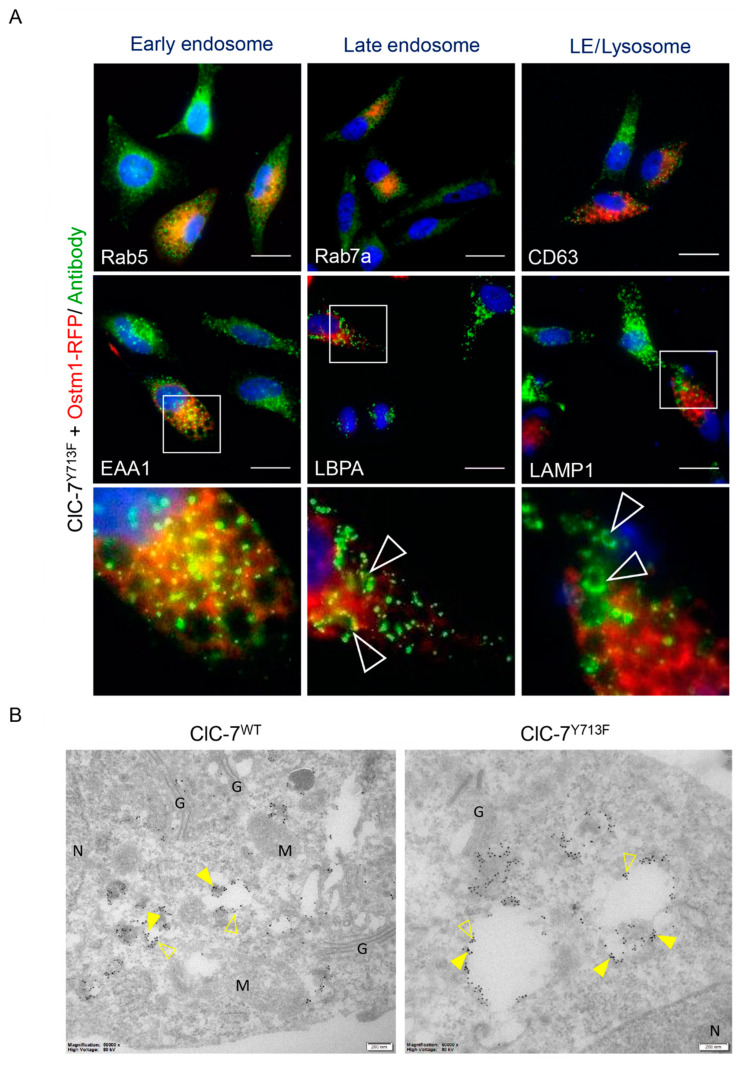
Enlarged compartments in ClC-7Y713F cells are of late endosomal/lysosomal nature. (**A**) Representative images of HeLa cells co-transfected with rClC-7Y713F and RFP-tagged Ostm1 (red) and immune-stained (green) with antibodies against early endosomes (Rab5, EEA1), late endosomes (Rab7a, LBPA) or lysosomes (CD63, LAMP1). Nuclei were stained with DAPI (blue). Scale bar, 25 µm. White arrowheads in the lowest panel, which displays magnifications of marked squares in images above, show enlarged vesicles positive for late endosomal and lysosomal markers. Single channels of insets (EEA1, LBPA and LAMP1) and equivalent areas (Rab5, Rab7a and CD63) are shown in Figure S1. (**B**) Immuno-EM of HeLa cells co-transfected with ClC-7 (WT or Y713F) and Ostm1-GFP and stained with anti-LAMP1 and anti-GFP. The enlarged compartments stain positive for both GFP (15 nm gold, exemplarily shown with filled arrowheads) and LAMP1 (10 nm gold, empty arrowheads) reinforcing their late endosomal/lysosomal nature. G—Golgi apparatus; M—mitochondria; N—nucleus. Scale bar, 200 nm.

To gain further insight into the composition of the enlarged vacuoles, we stained HeLa cells co-transfected with rClC-7^Y713F^ and Ostm1-RFP using antibodies against marker proteins of early endosomes (EEA1, Rab5), late endosomes (LBPA, Rab7a) and late endosomes/lysosomes (LAMP1, CD63) (Figure 3A and Figure S1). The enlarged vacuoles were expectedly positive for Ostm1-RFP, since ClC-7 requires Ostm1 for ion transport activity [3], and the vesicle enlargement did not occur in the absence of Ostm1 (Figure 1A). In turn, the presence of Ostm1-RFP on the enlarged vacuoles showed the presence of co-expressed rClC-7^Y713F^ because Ostm1 requires ClC-7 for ER export and lysosomal targeting [5,23]. The enlarged Ostm1-RFP-positive structures were positive for late endosomal and lysosomal, but not for early endosomal markers (Figure 3A). Immuno-EM confirmed the co-localization of Ostm1-GFP and LAMP1 on lysosomes in the case of co-expression with wild-type rClC-7 and on enlarged vesicles when co-expressed with rClC-7^Y713F^ (Figure 3B). These data showed that the enlarged vacuoles were derived from late endosomes and lysosomes.

### 3.4. The Enlarged Lysosomes Are Less Acidified

**Figure 4:**
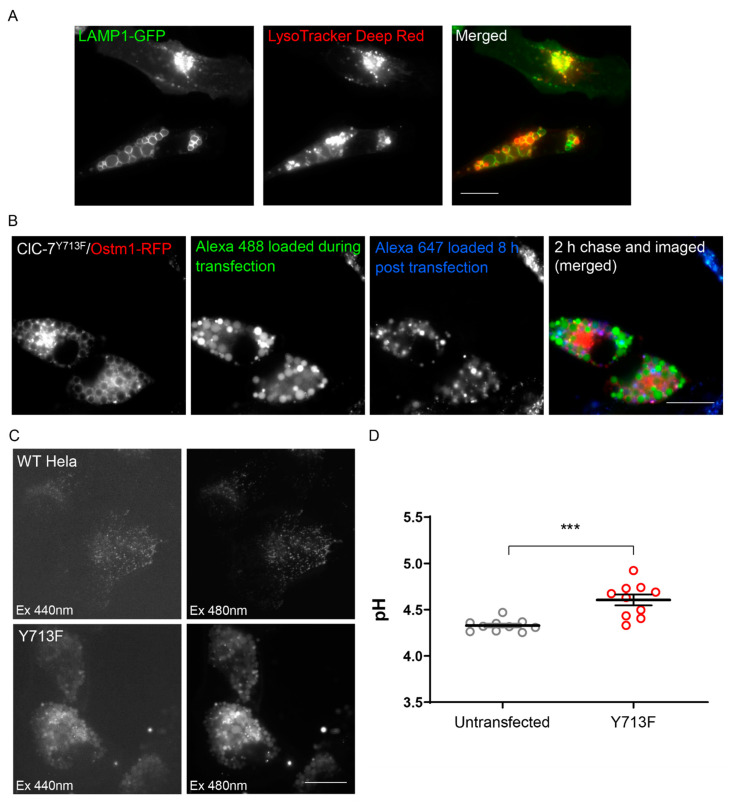
Enlarged endo-lysosomes are less acidic. (**A**) Localization of LysoTracker Deep Red in smaller vesicles compared with the larger vesicles suggesting a hypoacidic pH of the enlarged vesicles. Scale bar, 20 µm. (**B**) Lysosomal uptake of dextran-coupled fluorophores. Alexa 488 was applied during transfection and changed for Alexa 647 8 h after transfection. Scale bar, 20 µm. (**C**) Representative images showing uptake of dextran-coupled Oregon Green 488 in the lysosomal compartments of both untransfected (WT HeLa) and rClC-7^Y713F^/Ostm1-transfected (Y713F) cells. Scale bar, 20 µm. (**D**) Lysosomal pH in untransfected and ClC-7^Y713F^/Ostm1-transfected HeLa cells. Values represent mean ± s.e.m.; thick lines in data point clouds indicate the arithmetic mean, thin lines the s.e.m. (Student’s *t*-test significance shown as *** *p* < 0.0005) from 10 independent experiments with >10 cells per experiment and condition.

Expression of the ClC-7^Y715C^ mutant from patients was reported to lead to a hyperacidification of small lysosomes surrounding the enlarged vacuoles, whereas the vacuoles were not effectively acidified [20]. Consistently, we found that the LysoTracker Deep Red dye, which accumulates in acidic organelles, did not prominently stain the enlarged endo-lysosomes upon expression of rClC-7^Y713F^/Ostm1, while smaller vesicles within the same cells showed strong LysoTracker signal (Figure 4A). However, LysoTracker did stain the vacuoles, albeit weaker than smaller structures, mainly at the surrounding membrane rather than in the lumen. This staining was abolished by protonophore treatment, demonstrating its dependence on the transmembrane pH gradient (Figure S2). To quantitively measure the pH of the enlarged endo-lysosomes, we wanted to load them by endocytosis with the ratiometric pH-sensitive dye Oregon Green 488 coupled with dextran. However, the vacuoles in ClC-7^Y715C^-expressing cells from patients were reported to be almost inaccessible by endocytic cargo [20]. In agreement with this, we found that dextran-coupled Alexa dyes were not efficiently transported into pre-existing enlarged endo-lysosomes (Alexa 647 in Figure 4B). However, when we loaded lysosomes with dextran-coupled Alexa dye before the expression of rClC-7^Y713F^/Ostm1, the dye was found in the vacuoles after enlargement (Alexa 488 in Figure 4B). Hence, we could load these compartments with dextran-coupled Oregon Green 488 and measure luminal pH (Figure 4C). Measuring whole-cell fluorescence, we found a moderately increased pH (4.61 ± 0.06) in rClC-7^Y713F^/Ostm1-expressing cells in comparison with lysosomal pH (4.33 ± 0.02) in untransfected cells (Figure 4D). Measurement of the pH specifically in enlarged vacuoles (in 12 cells) by selecting their area manually revealed a similar moderately increased pH of 4.62 ± 0.10.

### 3.5. Impaired Degradative Property of the Enlarged Lysosomes

**Figure 5:**
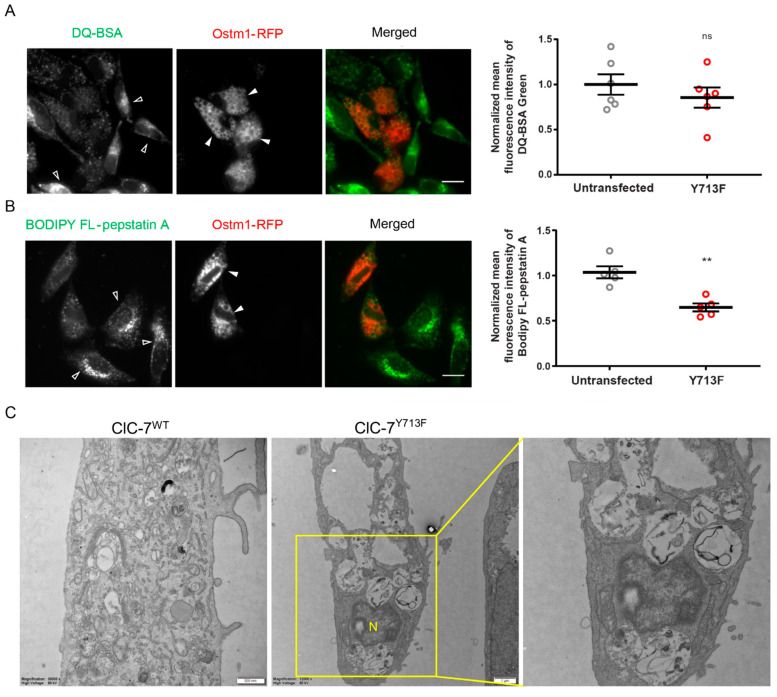
Degradation defect in the enlarged lysosome. (**A**) Representative fluorescence images of ClC-7^Y713F^/Ostm1-RFP-expressing (filled arrowheads) and non-expressing cells (empty arrowheads) 1 h after DQ-BSA application. Scale bar, 20 µm. Right, mean ± s.e.m where thick lines in data point clouds indicate the arithmetic mean and thin lines the s.e.m., N = 6. Statistical significance tested by Student’s *t*-test: ns—not significant. (**B**) Representative fluorescence images of HeLa cells co-transfected with ClC-7^Y713F^/Ostm1-RFP (red) loaded with BODIPY FL-pepstatin A (green) for 1 h to assess cathepsin D activity. BODIPY FL-pepstatin A signal appears stronger in untransfected (empty arrowheads) than in transfected (filled arrowheads) cells. Scale bar, 20 µm. Right, mean ± s.e.m. from 5 independent experiments with >40 cells analyzed per condition. Statistical significance by Student´s *t*-test shown as ** *p* < 0.005. (**C**) Electron micrographs of Epon sections with electron-lucent swollen vacuoles in cells transfected with ClC-7^Y713F^/Ostm1 (middle and enlargement on right) but not with ClC-7^WT^/Ostm1 (left). Scale bars, 0.5 µm for ClC7^WT^ and 1.0 µm for ClC-7^Y713F^.

The degradative capability of the enlarged lysosomes was checked using dye-quenched bovine serum albumin (DQ-BSA), whose proteolysis results in dequenching and increased fluorescence of the attached self-quenching dye BODIPY FL. We found a trend but no significant reduction in BODIPY FL fluorescence upon expression of ClC-7^Y713F^/Ostm1-RFP in comparison with untransfected cells (Figure 5A). To specifically assess the enzymatic activity of the lysosomal protease cathepsin D, we used BODIPY FL-pepstatin A, which binds to the active site of cathepsin D and whose fluorescence is an indicator of cathepsin D activity [30]. BODIPY fluorescence in ClC-7^Y713F^/Ostm1-RFP-expressing cells was significantly reduced by about 35% in comparison with untransfected cells (Figure 5B). This reduction was not due to a decrease in protein levels of mature cathepsin D (Figure S3). Consistent with a diminished degradative activity of the enlarged lysosomes, electron microscopy revealed that the vacuoles in ClC-7^Y713F^/Ostm1-transfected cells were generally electron-lucent, with an accumulation of materials at distinct stages of degradation, indicating a build-up of lysosomal storage (Figure 5C).

### 3.6. Autolysosomal Nature of the Enlarged Vesicles

**Figure 6:**
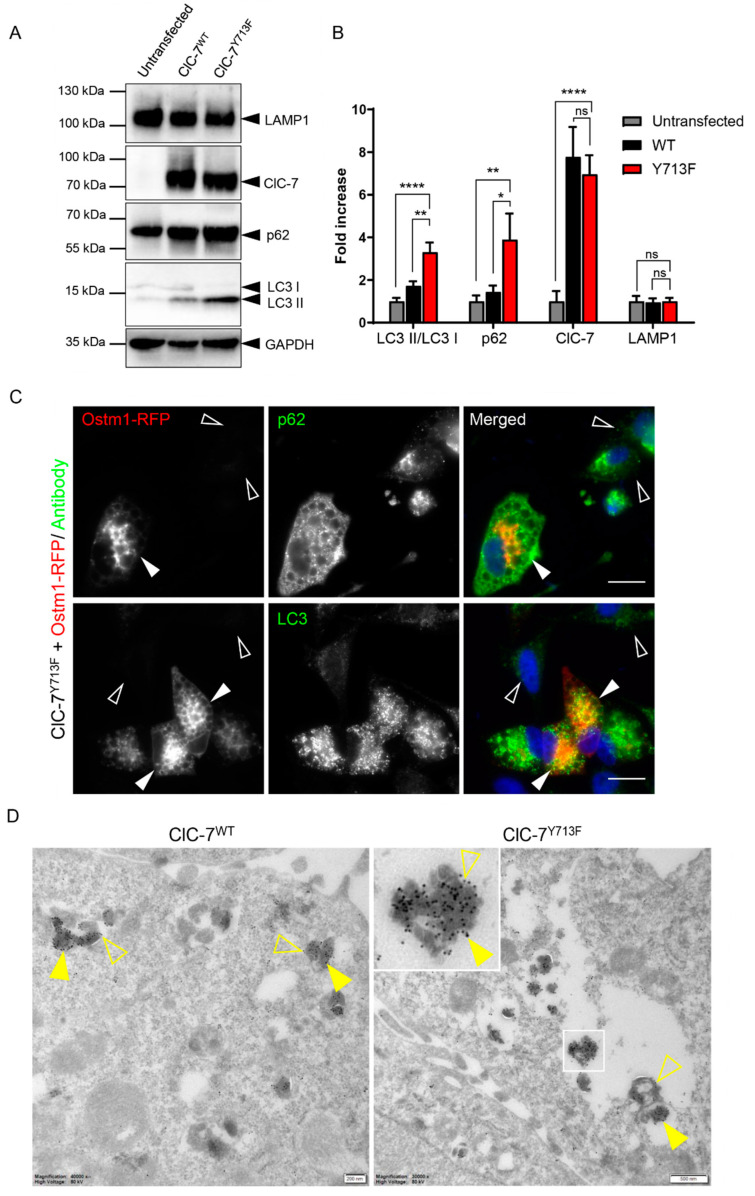
Enlarged compartments can be of autolysosomal nature. (**A**) Representative immunoblot of proteins from HeLa cells, either untransfected or transfected with ClC-7^WT^/Ostm1 or ClC-7^Y713F^/Ostm1, with antibodies against ClC-7, the lysosomal marker protein LAMP1, autophagy-related proteins p62 and LC3 and GAPDH as loading control. Original blot images can be found in Figure S4. (**B**) Quantification of immunoblotting as in (**A**). Protein levels of autophagy markers SQSTM1/p62 and the LC3-II/LC3-I ratio were increased in ClC-7^Y713F^/Ostm1-transfected cells compared with ClC-7^WT^/Ostm1-transfected and untransfected cells, whereas LAMP1 expression remained unchanged. Values represent mean ± s.e.m from >4 independent experiments each. Statistical significance assessed by one-way ANOVA with Tukey’s multiple comparison test for each condition is indicated as * *p* < 0.1, ** *p* < 0.01, **** *p* < 0.0001 or ns for not significant. (**C**) Immuno-staining of HeLa cells co-transfected ClC7^Y713F^/Ostm1-RFP (red) against autophagic markers p62 and LC3 (green), which showed stronger punctate signal in transfected (filled arrowheads) than in untransfected (empty arrowheads) cells. Scale bar, 20 µm. (**D**) Electron micrographs of ultrathin cryosections of HeLa cells co-transfected with ClC-7, either WT or Y713F, and Ostm1-GFP. Cells were labeled for GFP (15 nm gold, exemplarily shown with filled arrowheads), present on the limiting membrane of late endosomes/lysosomes, and p62 (10 nm gold, exemplarily shown with empty arrowheads). Scale bars, 200 nm for ClC-7^WT^ and 500 nm for ClC-7^Y713F^.

As lysosomal dysfunction is likely to affect autophagosome clearance, we tested for an effect of the ClC-7 mutant on the abundance of autophagy markers LC3 and p62. Western blotting revealed a two- to three-fold increase in the LC3 II/LC3 I ratio upon transfection of rClC-7^Y713F^/Ostm1 compared with rClC-7^WT^/Ostm1-transfected or untransfected HeLa cells, while transfection of rClC-7^WT^/Ostm1 did not significantly alter the LC3 II/LC3 I ratio (Figure 6A,B). The protein levels of p62, also named sequestosome 1 (SQSTM1), were similarly affected. The combination of an increased LC3 II/LC3 I ratio and an increase in the protein levels of p62 suggested reduced clearance of autophagosomes [31,32,33,34,35]. In contrast, the amount of the lysosomal marker protein LAMP1 was not altered (Figure 6A,B), suggesting that the morphological alterations of lysosomes were not accompanied by increased levels of lysosomal proteins. Immunostaining revealed an accumulation of punctate structures positive for LC3 and p62 in cells transfected with rClC-7^Y713^/Ostm1-RFP (Figure 6C), consistent with a possible defect in the clearance of autophagic material. Indeed, immuno-EM showed an accumulation of p62 in the enlarged vesicles in rClC-7^Y713^/Ostm1-GFP-expressing cells, identifying them as autolysosomes (Figure 6D). These inclusions of p62 aggregates, also known as p62 bodies, are a hallmark of impaired autophagic clearance [36,37].

## 4. Discussion

### 4.1. ClC-7 Gain-of-Function Leads to Altered Gating Kinetics and Lysosomal Morphology

The mutation of a tyrosine in the CBS domain of ClC-7 leads to a gain-of-function of this Cl^−^/H^+^ exchanger [20]. The increase in ClC-7 currents is explained by a loss of an inhibitory interaction with the lipid PI(3,5)P_2_, resulting in a constitutively active antiporter [21]. In the resolved structure of ClC-7, a non-protein density could be modeled as a bound phosphoinositol-3-phosphate (PI3P) [6]. The tyrosine, with its hydroxyl group at the large phenyl ring, is shown to be involved in the binding of PI3P or PI(3,5)P_2_, and its substitution by any other tested amino acid, including cysteine and phenylalanine, results in increased ClC-7 currents, supposedly due to reduced PI(3,5)P_2_ binding [21]. Like wild-type ClC-7, the tyrosine mutant is still activated by low extracellular Cl^−^ [38]. The increase in ClC-7 activity in turn is thought to underlie the formation of large intracellular vacuoles in cells of patients and mice with a mutation in this tyrosine [20]. In agreement with this notion, we find that co-expression of Ostm1, which is required for ion transduction by ClC-7 [3], is also required for the enlargement of lysosomes. Additionally, we show that combining the tyrosine mutation with a mutation of the proton glutamate (E312A), which strongly diminishes ion transport [3,28,29], prevents vacuolization. Furthermore, lysosomes are not enlarged when the tyrosine mutation is combined with a mutation of the gating glutamate (E245A), which uncouples chloride from proton transport [3,14]. This demonstrates that not only Cl^−^ transport but specifically Cl^−^/H^+^ exchange by ClC-7 is required to cause vacuolization.

Organelles in the endo-lysosomal pathway adapt shape and size according to osmotic forces [39,40]. Enlargement is seen when export of metabolites is impaired [41,42,43] or upon dilution of the cytosol during osmotic cell swelling in a hypotonic environment [44,45]. Cl^−^/H^+^ exchange by ClC-7 leads to a pH gradient-driven accumulation of luminal Cl^−^ [14]. Therefore, increased transport of chloride into the lysosome due to the gain-of-function mutation in ClC-7 would lead to increased osmotically driven influx of water and organelle swelling [20]. In agreement with this, we find that the combination of the tyrosine mutation with the gating glutamate mutation (E245A), which reduces lysosomal Cl^−^ accumulation [14], does not cause the formation of large endo-lysosomal vacuoles. Indeed, the enlarged vacuoles appear to be osmotically swollen. As live-cell imaging of fusion events between enlarged vesicles shows, the fused compartments immediately acquire a round shape. This has previously also been shown for gain-of-function mutations in the late endosomal ClC-6 [46]. In both cases, this is indicative of osmotic water influx. Further studies, such as measurements of luminal chloride concentration, are required to test the notion that ClC-7 gain-of-function accumulates excessive chloride in lysosomes leading to their osmotic swelling.

However, an increase in the osmotic potential alone is unlikely to explain the drastic vacuolization, as it also requires a considerable additional amount of limiting membrane. Furthermore, osmotically swollen lysosomes are usually not as drastically enlarged as with this gain-of-function ClC-7 mutant [39,40]. Altered membrane tension upon osmotic swelling may affect fusion and fission processes of these compartments [39,40]. Ion and osmolyte efflux allows for volume decrease and hence reduced membrane tension. This is crucial for tubulation and vesiculation to reconstitute lysosomes after homotypic or heterotypic fusion events. Impairing fission without affecting fusion would lead to an enlargement of the respective compartment. One process that relies on tubulation and membrane fission is autophagic lysosome reformation (ALR) [47]. Consistent with impaired ALR, we find that the enlarged vacuoles are indeed autophagosomes. Notably, inhibition of PIKfyve, the phosphoinositide kinase that generates PI(3,5)P_2_, which is shown to inhibit ClC-7 activity [21], also results in an enlargement of lysosomes and accumulation of autophagic material [48]. However, in that case, the autophagic material does not co-localize with the enlarged vacuoles, suggesting that PIKfyve inhibition obstructs heterotypic fusion between autophagosomes and lysosomes [48], whereas this process is not prevented by the gain-of-function ClC-7^Y713F^ mutant.

### 4.2. ClC-7 in Lysosomal pH and Ion Homeostasis

As electrogenic chloride transporters in the endosomal/lysosomal pathway, CLCs can provide the countercharge for proton import by the V-ATPase to support luminal acidification [49]. However, the role of ClC-7 in regulating lysosomal pH has been debated. Steady-state lysosomal pH is unchanged in various cell types upon loss of ClC-7/Ostm1-deficient mice [5,9,14,50] or its *C. elegans* ortholog Clh-6 [15,17], and cation counter-flux suffices in supporting lysosomal acidification [50]. Nicoli et al. report that, while the enlarged intracellular vacuoles are not acidified as judged by the lack of LysoTracker staining, surrounding smaller lysosomes are hyperacidified, consistent with a gain of ClC-7 function that enhances lysosomal acidification [20]. However, our electron micrographs of ClC-7^Y713F^-expressing cells do not confirm the presence of lysosomes with regular size surrounding the enlarged vesicles. Confirming the observation by Nicoli et al. [20], we also observe that the vacuoles are in general not efficiently stained by LysoTracker. In our hands, some vacuoles are clearly positive for LysoTracker, whereas most others show weak pH gradient-dependent LysoTracker signal. When we measure the pH of the enlarged compartments after endocytic loading with the ratiometric dye Oregon Green 488, we find a moderately alkalinized pH in ClC-7^Y713F^-expressing cells compared with lysosomes of untransfected cells. The reason for the apparent discrepancy between the strong reduction in LysoTracker signal with only a moderate effect on luminal pH (0.3 pH unit increase) is unclear. Notably, the vacuoles are not equally loaded with endocytosed dye. If more acidic vacuoles had taken up more Oregon Green 488-dextran, this would shift the apparent overall pH value to more acidic pH. The large vacuoles, which fill almost the entire cytoplasm upon transient transfection with the gain-of-function ClC-7^Y713F^ mutant, are efficiently loaded with Oregon Green 488-dextran. This prevents the specific measurement of the pH of normal-sized lysosomes that are reported to be hyperacidified [20]. While the impact of the ClC-7 gain-of-function mutation on lysosomal pH remains to be clarified, it has been shown previously that the enlargement of the endo-lysosomal compartments is not strictly correlated with alterations in luminal pH [21].

Altered Cl^−^ transport is likely to affect further parameters within the complex system of lysosomal ion homeostasis, such as the lysosomal membrane potential and Ca^2+^ [51,52,53]. Efflux of luminal Ca^2+^ plays a role in several lysosomal functions, including induction of autophagy and regulating lysosomal fusion and fission with other cellular organelles like autophagosomes, endosomes and the plasma membrane [54,55,56]—processes whose dysfunction may contribute to the formation of the large intracellular vacuoles observed with the gain-of-function of CLC-mediated Cl^−^/H^+^ exchange.

### 4.3. Reduced Lysosomal Degradation and Autophagic Clearance

Reduced activity of cathepsin B has previously been shown for the enlarged lysosomes with the gain-of-function ClC-7^Y715C^ patient mutant [20]. Accordingly, we find a tendency of reduced protein degradation upon expression of rClC-7^Y713F^. It has recently been shown that reduced luminal Cl^−^ decreases the activity of particular lysosomal enzymes like cathepsin C, lysozyme and DNase II [16,17]. As ClC-7 is crucial for luminal accumulation of Cl^−^ [14], this may at least in part explain the reduced degradative capacity of ClC-7-deficient lysosomes [13]. However, the gain-of-function mutation of ClC-7 is unlikely to result in reduced luminal [Cl^−^]. Therefore, the diminished lysosomal degradation may rather be due to altered trafficking events that are affected by alterations in vesicular Cl^−^ homeostasis and accompany the vacuolization [51] or directly by the moderately increased pH seen here with heterologous expression of the gain-of-function ClC-7 mutant. Depending on the metabolic state of the cell, lysosomes are acidified to a pH of ~4.5, at which lysosomal enzymes are most active [49,57,58], and an increase in pH by 0.2 units can indeed correlate with a significant reduction in lysosomal protein degradation [58].

We find a marked increase in autophagy-related proteins in ClC-7^Y713F^-expressing cells compared with ClC-7^WT^. Furthermore, our immuno-EM data reveal the presence of dense aggregates of p62 in ClC-7^Y713F^-transfected cells, indicative of a block of autophagic clearance [33,59], which is in agreement with the reduced lysosomal degradative capacity. Similarly, an accumulation of autophagic material is also found in ClC-7-deficient tissue with impaired lysosomal degradation and upon ClC-7 knockdown in cardiomyocytes [13,60].

On the other hand, bone resorption by osteoclasts does not seem to be affected by the ClC-7^Y715C^ mutant, as no osteopetrosis is reported for patients and the mouse model with this ClC-7 variant [20]. This suggests that lysosomal fusion with the plasma membrane, which is responsible for the formation of the ruffled border in osteoclasts, remains unaffected by the presumed increase in lysosomal osmotic pressure caused by ClC-7 gain-of-function. Conversely, ClC-7 loss-of-function results in an underdevelopment of the ruffled border [1,14,29]. While enhanced ClC-7 activity impacts lysosomal ion balance, osmotic equilibrium and likely membrane potential [14,53], it may lack similar effects on the extracellular resorption lacuna of osteoclasts.

## 5. Conclusions

So, while loss-of-function and gain-of-function mutations in ClC-7 can cause diverse clinical phenotypes, such as osteopetrosis, neurodegenerative lysosomal storage disease, myelination defects and albinism, their pathomechanisms share an impairment of lysosomal function and autophagic flux.

## Data Availability

All experimental data can be found in the figures presented in this manuscript.

## Supplementary Materials

The following supporting information can be downloaded at: https://www.mdpi.com/article/10.3390/biom13121799/s1, Figure S1: Magnifications of images shown in Figure 3A; Figure S2: LysoTracker staining of enlarged vacuoles; Figure S3: Expression levels of cathepsin D; Figure S4: Original blot images; Video S1: Fusion of LAMP1-positive vacuoles.
