# Supplementary material for: Impaired Autophagic Clearance with a Gain-of-Function Variant of the Lysosomal Cl−/H+ Exchanger ClC-7
Source: Biomolecules. 2023 Dec 15;13(12):1799. doi: 10.3390/biom13121799 (PMC10742274; doi:10.3390/biom13121799)
Supplement: Supplementary file 1 [file biomolecules-13-01799-s001.zip › Bose_CLC7_suppl.pdf]

Supplementary Figures to

Impaired autophagic clearance with a gain-of-function variant of the lysosomal Cl^-/H^+ exchanger ClC-7

by

Shroddha Bose, Cecilia de Heus, Mary E. Kennedy, Fan Wang, Thomas J. Jentsch, Judith Klumperman and Tobias Stauber

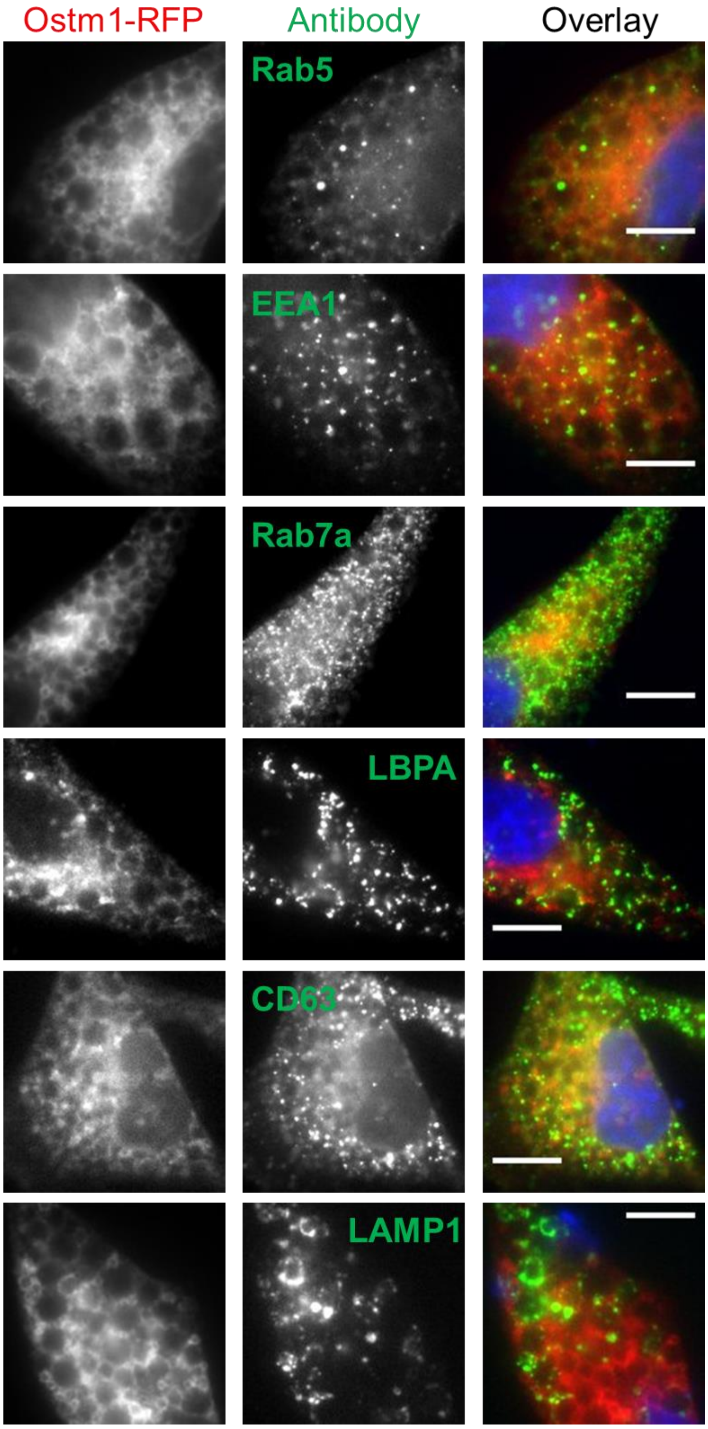

Figure S1. Magnifications of images shown in Figure 3A of HeLa cells co-transfected with rClC-7^{Y713F}/Ostm1-RFP (red in overlay) and immune-stained (green in overlay) with antibodies against early endosomes (Rab5, EEA1), late endosomes (Rab7a, LBPA) or lysosomes (CD63, LAMP1). Nuclei were stained with DAPI (blue in overlay). Scale bar, 10 μm.

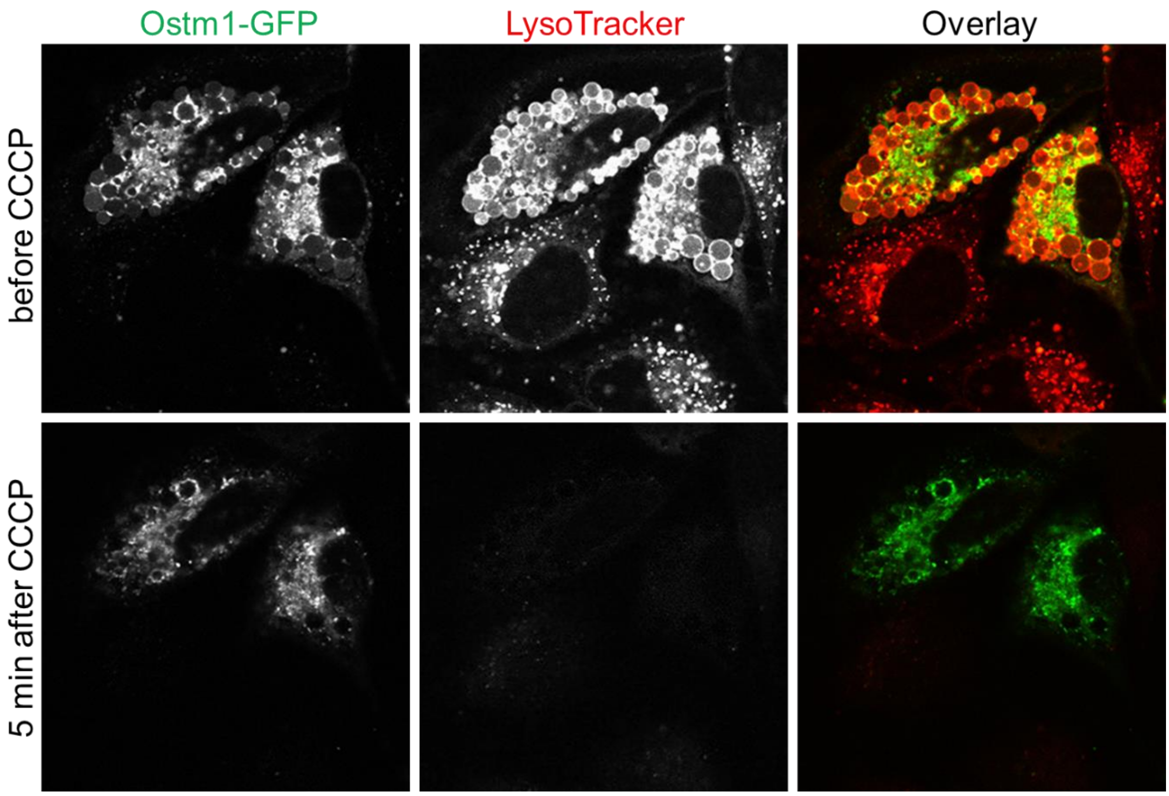

Figure S2. LysoTracker staining of enlarged vacuoles. HeLa cells were stained with LysoTracker (red in overlay) 16 h after transfection with rClC-7^{Y713F}/Ostm1-GFP (green in overlay) and imaged before (upper panel) and 5 min after (lower panel) application of the protonophore carbonyl cyanide 3-chlorophenyl hydrazine (CCCP) at 200 μM.

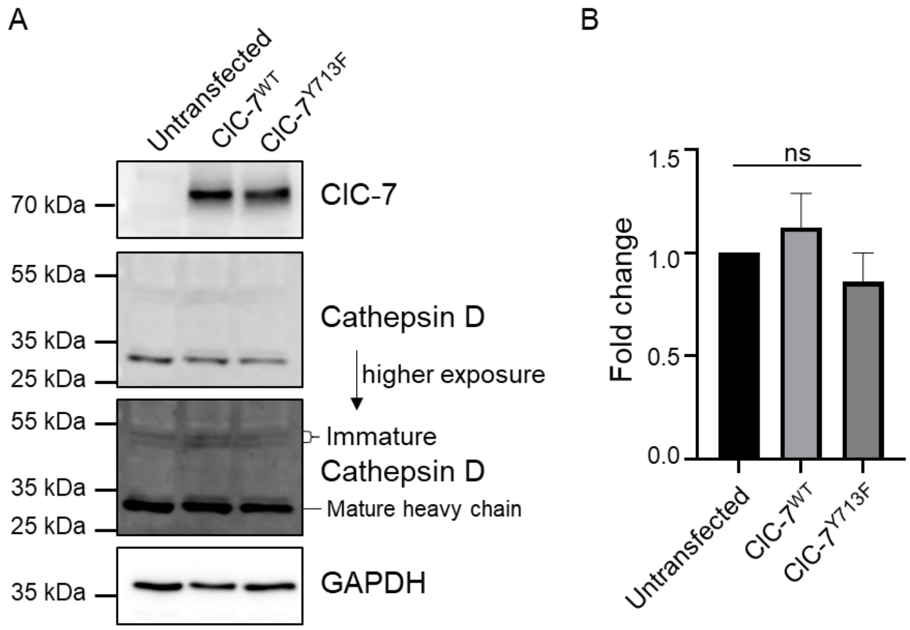

Figure S3. Expression levels of cathepsin D. **(A)** Representative immunoblot of proteins from HeLa cells, either untransfected or transfected with rClC-7^{WT}/Ostm1-RFP or rClC-7^{Y713F}/Ostm1-RFP, with antibodies against ClC-7, cathepsin D, and GAPDH as loading control. **(B)** Quantification of immunoblotting as in (A). Protein levels of the mature heavy chain of cathepsin D were not altered in ClC-7^{WT}/Ostm1- or ClC-7^{Y713F}/Ostm1- transfected cells. Values represent mean ± s.e.m from 4 independent experiments. Statistical significance assessed by one-way ANOVA with Tukey's multiple comparison test showed no significance (ns) with $p > 0.1$.

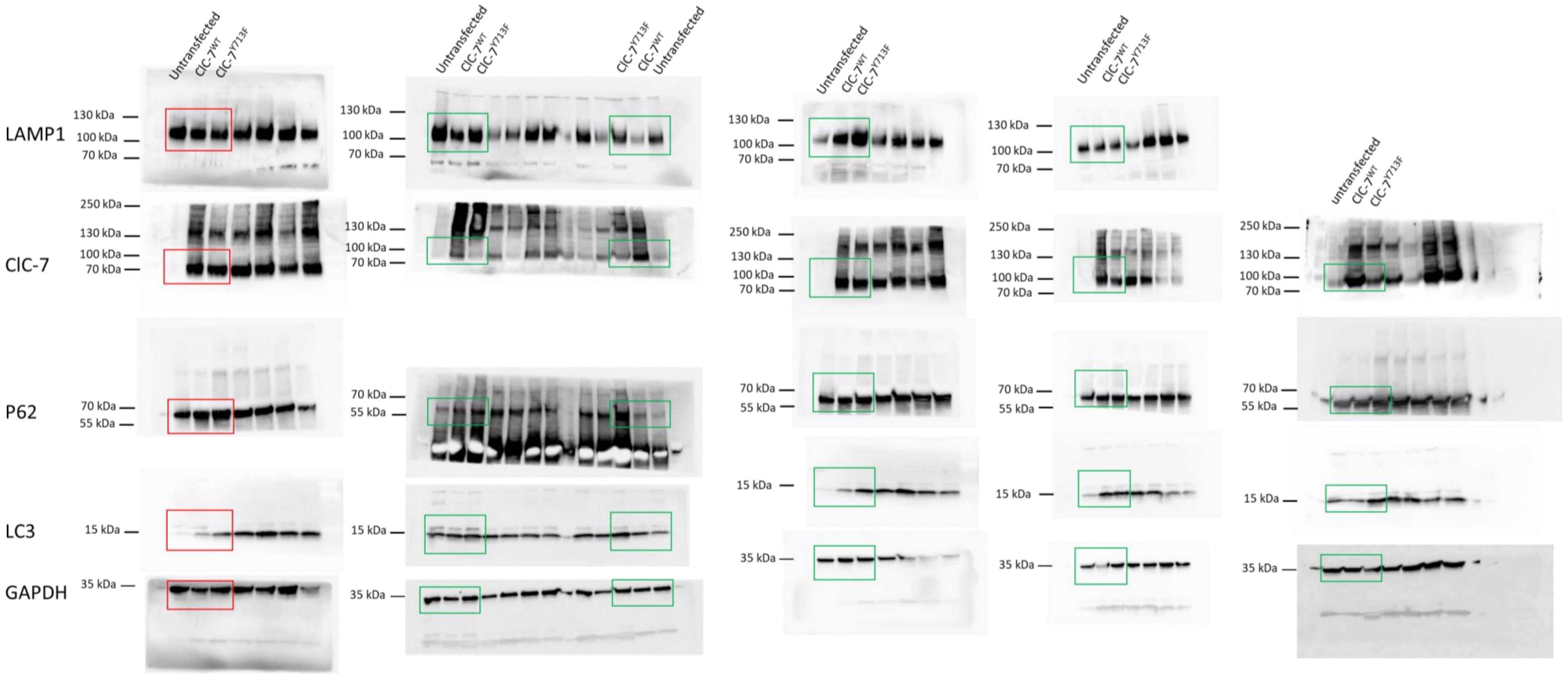

Figure S4. Original blot images. Red boxes mark the cropped regions shown in Figure 6A. Green boxes, independent biological replicates used for quantification in Figure 6B.
